# FE-learning and the virtual transformation of histopathology teaching during COVID-19: its impact on student learning experience and outcome

**DOI:** 10.1186/s12909-021-03066-z

**Published:** 2022-01-07

**Authors:** Samantha Waugh, James Devin, Alfred King-Yin Lam, Vinod Gopalan

**Affiliations:** School of Medicine & Dentistry, Griffith University, Gold Coast, QLD 4222 Australia

**Keywords:** COVID-19, Pathology, Histopathology, Education, Medical, Online learning

## Abstract

**Background:**

Medical and pathology education has gone through an immense transformation from traditional face-to-face teaching mode to virtual mode during the COVID-19 pandemic. This study evaluated the effectiveness of online histopathology teaching in medical education during the 2020 COVID-19 pandemic in Griffith University, Australia.

**Methods:**

Second-year medical students (*n* = 150) who had previously completed one year of face-to-face histopathology teaching, completed an online questionnaire rating their learning experiences before and during the COVID-19 pandemic after the completion of their histology and pathology practical sessions. The students' histopathology assessment results were then compared to the histopathology results of a prior second-year cohort to determine if the switch to online histopathology teaching had an impact on students' learning outcome.

**Results:**

A thematic analysis of the qualitative comments strongly indicated that online histopathology teaching was instrumental, more comfortable to engage in and better structured compared to face-to-face teaching. Compared to the previous year's practical assessment, individual performance was not significantly different (*p* = 0.30) and compared to the prior cohort completing the same curriculum the mean overall mark was significantly improved from 65.36% ± 13.12% to 75.83% ± 14.84% (*p* < 0.05) during the COVID-19 impacted online teaching period.

**Conclusions:**

The transformation of teaching methods during the 2020 COVID-19 pandemic improved student engagement without any adverse effects on student learning outcomes in histology and pathology education.

## Background

Pathology teaching is an essential component of pre-clinical medicine. It provides the pillars for understanding disease aetiology and pathogenesis, which is the basis of all diagnosis and therapy [1, 2]. Junior doctors must understand the science underpinning disease processes in order to explain the nature of the disease to a patient and to understand and use the language of medicine [1].

Over the years, changes to pathology education have been adapted to reflect the expansion of medical knowledge, increased student numbers, and technological innovations. Pathology education typically consists of a combination of teaching methods, including lectures, tutorials, and practicals. Exposure to traditional microscopes facilitates interactive learning and provides moving imprints of tissues compared to static images from textbooks or gross pathological specimens [3]. This enables students to identify, zoom in on and study various tissue samples. More recently, a combined didactic method integrating components of virtual microscopy with face-to-face teaching and conventional light microscopy has been implemented [4]. This has improved student interactions and pathology learning experiences by increasing student curiosity and implementing basic science related clinical casesto improve understanding [5].

COVID-19 presented an unprecedented challenge to both pathology education and medical education as a whole. Traditionally, pathology teaching relied on face-to-face contact using gross pathological specimens, conventional light microscopy, and the integration of other biomedical disciplines such as anatomy, radiology and pathophysiology. This no longer became feasible during university closures in response to the global pandemic. Despite the implementation of virtual microscopy, digital image/audio modules and podcasts for gross pathology [6, 7], face-to-face teaching has always been a core component of pathology education [1].

The uncertainty surrounding the COVID-19 pandemic called for an innovative approach to support ongoing medical education and pathology teaching despite lockdown restrictions. Therefore, a unique opportunity presented itself to completely transform face-to-face teaching to an online mode of delivery for pathology education to overcome this challenge. In this study, we aimed to evaluate the effectiveness of online teaching for pathology to ensure that medical students learning outcomes were met and that pathology education was able to continue.

## Methods

### Student groups

Students (*n* = 220) enrolled in the second year of the Doctor Medicine (MD) programme at Griffith University, Australia was asked to participate in this study. All second-year students across both campuses (Gold Coast and Sunshine Coast) were invited to participate in the online questionnaire. The research methodology and approaches were previously approved by the Griffith University Human Research Ethics committee (GU ref no: 2018/928).

### Study design

An observational case–control study design was used where the second-year medical students who had previously completed one year of face-to-face histopathology teaching were used as both cases (during COVID) and controls (pre-COVID). After completing their histology and pathology practical sessions, these students have completed an online questionnaire rating their learning experiences before and during the COVID-19 pandemic.

**Fig. 1:**
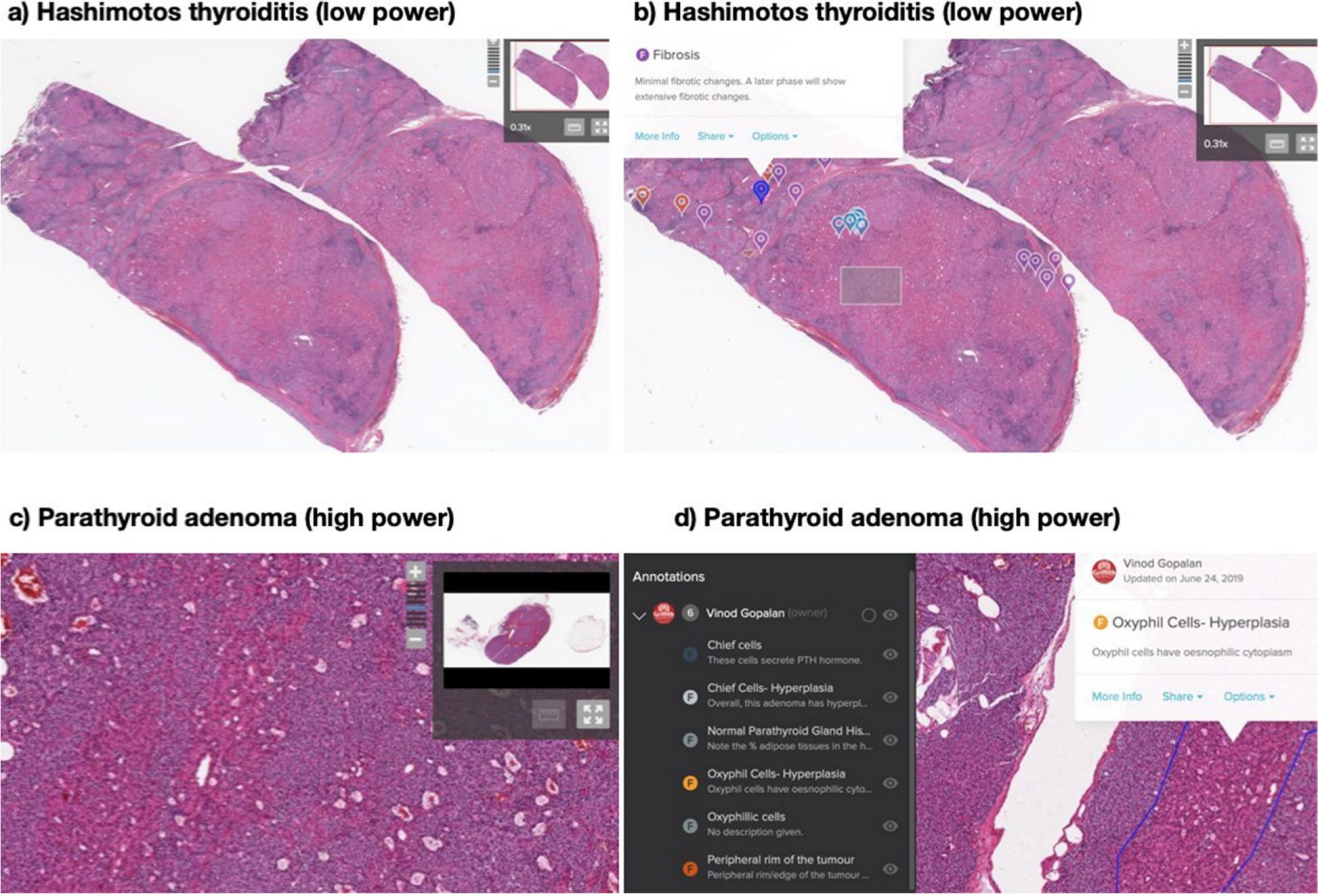
Representation of the online BEST pathology slides. **a** Hashimoto's thyroiditis in low power without any annotation; **b** Annotations highlighting the key histopathological features such as fibrosis and inflammatory cell infiltration; **c** Parathyroid adenoma in high power without any annotations; **d** Annotations showing oxyphil cell hyperplasia

To replicate face-to-face lectures and practical sessions, various online classrooms were created. Blackboard Collaborate Ultra, an e-learning platform where instructors can host live chat sessions, was used to create virtual live classrooms for lecture delivery. Virtual microscopy to replicate conventional microscopy was incorporated into these online sessions (Fig. 1a-d). To replicate face-to-face practical sessions, tutor-assisted classrooms were created in smaller groups via Microsoft Teams.

Electronic slides were made available to students prior to and during practical sessions through BEST slice, a cloud-based library of high-resolution biomedical histology images [8] (Bao 2020). A practical handout and self-assessment notes were provided electronically to facilitate online discussions with reference to key pathological findings on the e-slides or gross pathology specimens. All of these sessions were delivered during the academic period of the second year MD programme. Each virtual lecture was delivered in a 2-h session with the entire year 2 cohort. The cohort was divided into six groups for each virtual practical, each group hosted by a student tutor over a 2.5-h session. Groups remained the same for each practical but were rotated through each tutor to ensure fair exposure to different tutoring styles. Additionally, tutors were monitored for consistency in teaching.

### Student evaluation and analysis

All students were invited to complete an online questionnaire (Table 1) rating the value of their online learning experience after completion of their final practical. All responses to the questionnaire were anonymous. Each of the seven questions was ranked on a scale between 1 and 5 (with 1 being strongly disagree and 5 being strongly agree). Questions evaluated students perception of online teaching as compared to traditional face-to-face teaching. Open responses regarding the effectiveness of online teaching were also recorded (Table 2).

**Table 1:** Student ratings of their learning experience in pathology before and after the switch to online learning

Student Rating (1, strongly disagree; 5, strongly agree)							
**Question**	**1**	**2**	**3**	**4**	**5**	**Mean ± SD**	**Median**
The switch to online pathology learning during COVID-19 was beneficial to my learning	4	3	10	51	83	4.36 ± 0.898	5
The online pathology practicals and lectures were as effective and engaging as face-to-face teaching	2	10	9	50	79	4.29 ± 0.945	5
The self-assessment questions were better explained in online sessions	2	3	11	33	101	4.48 ± 0.848	5
I feel more confident in learning micro- and macro-scopic structures with the online learning compared to face-to-face teaching	3	11	23	51	62	4.05 ± 1.022	4
The online pathology delivery was more beneficial in integrating the subject with other disciplines than face-to-face teaching	2	9	33	44	62	4.03 ± 0.999	4
Compared to other aspects of medical school, the change to online pathology teaching has been easier and is more beneficial	1	3	14	53	79	4.37 ± 0.790	5
Online pathology teaching should continue next year	6	8	14	50	72	4.16 ± 1.062	4

**Table 2:** Qualitative open responses received during the questionnaire

POSITIVE	NEGATIVE
I learned more during one online pathology practical than I did with all the in person practicals combined	Face-to-face lectures have a better flow and are easier to stay engaged with
Really valuable	I don’t personally believe that the learning experience can be replicated online
Would strongly recommend delivering the pathology content online to years 1 and 2 in future	In person labs have more individual attention per student
Very beneficial and in some instances more effective than face-to-face sessions	Lectures should stay face-to-face
I feel like I have a good grasp on pathology for the first time in my life	Much better mode of learning due to lack of room in face-to-face labs
A more efficient way to learn material	No experience using a microscope
I understand pathology much better and have gotten a lot more out of the pracs	Self-directed learning was easier face-to-face
During most of the in-person lab there was no structure and this did not help with learning	
I find myself a lot more engaged in learning pathology	
I have felt a lot more supported online and I feel more confident compared to last years face-to-face sessions	
Online histology and pathology was significantly more valuable	
Online teaching would be highly beneficial to future cohorts	
The depth of explanation was better online than face-to-face	
During most of the in-person lab there was no structure and this did not help with learning	

**Fig. 2:**
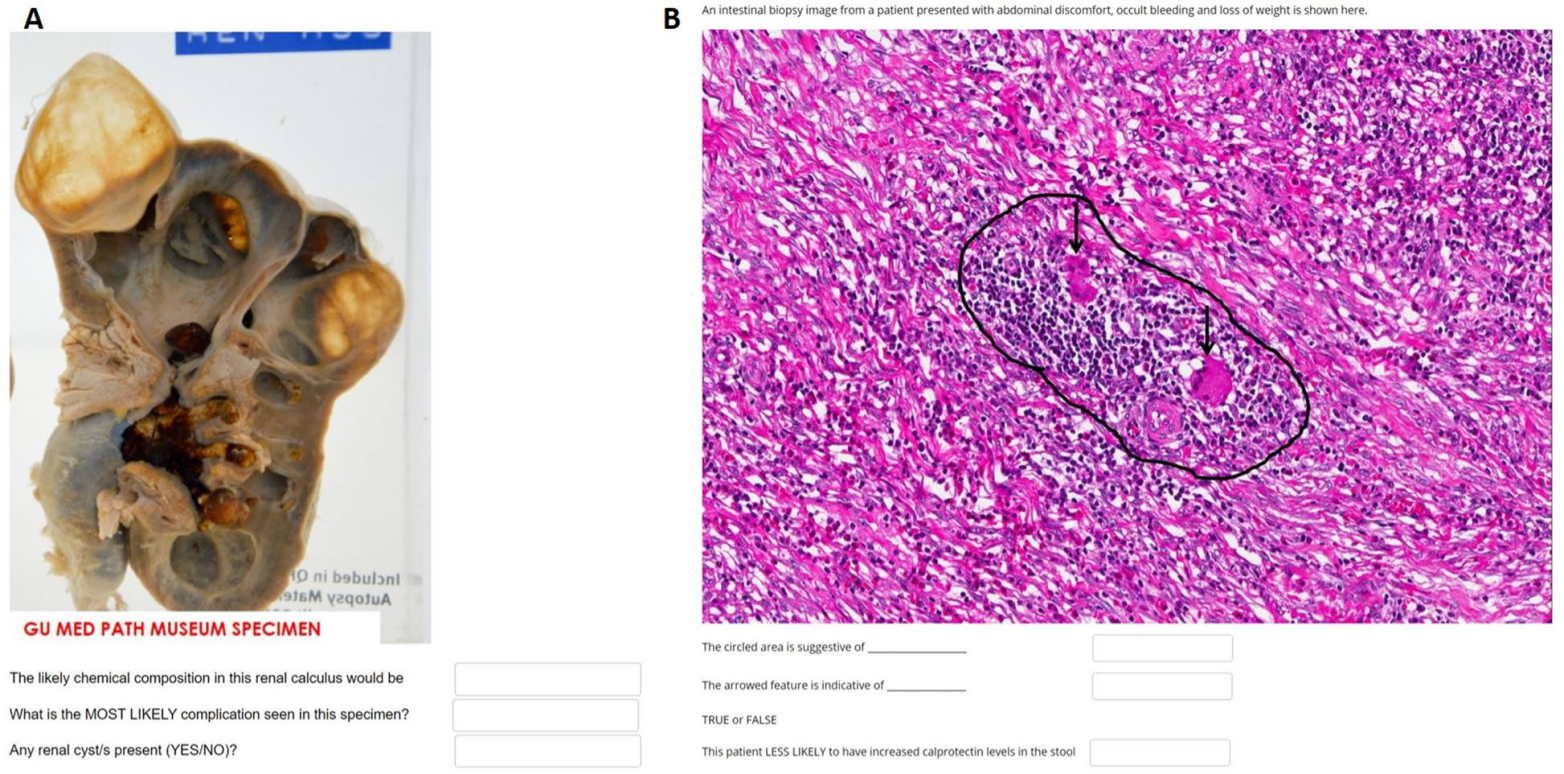
Examples of online practical assessments using both (**a**) gross pathology and (**b**) histopathology specimens

Student performance was assessed using scores achieved during the end of year assessment within the pathology discipline. In 2019, the practical assessment included stations containing three related questions about a particular topic (Fig. 2a and b) linked to discrete practical sessions studied throughout the year. The practical assessment in 2020 was conducted using a similar structure in an online format. Students were required to sign an academic integrity declaration prior to sitting the exam and a proctoring service was used online to track student response times and flag significant changes to usual student performance to prevent and detect cheating. Each individual station is standardised with a mark that is considered the expected standard (the sum of minima). These standards were compared between 2019 and 2020 to determine the likeness of the exam difficulty. Scores were compared between the second-year cohorts of 2019 and 2020 for differences in average performance. Scores were also compared at an individual level for students between the year 1 (standard, face to face learning) and year 2 cohorts (online curriculum) to assess for any changes in performance within the cohort following the transition to online learning.

Statistical analysis was completed using the Mann–Whitney test and the Wilcoxon matched-pairs signed rank test for unpaired and paired analysis, respectively. Non-parametric analysis based on the non-normal distribution of the data according to the Shapiro–Wilk test. Statistical analysis was completed using GraphPad Prism 8 (La Jolla, CA) analysis software.

## Results

### Student evaluation: learning and engagement

In total 150 students completed the online questionnaire. Students greatly appreciated the switch to online teaching during COVID-19 and found it not only highly beneficial for their learning (mean 4.36/5) but largely agreed (86%, 129/150) that online teaching was as effective and engaging as traditional face-to-face teaching (Table 1). The majority of students agreed (89.3%, 134/150) that self-assessment questions were learned better in online practicals compared to previous face-to-face sessions. Further, 74% (111/150) of students agreed that they now feel more confident in understanding tissue changes in pathological conditions after receiving the online lectures and practical sessions. Qualitative comments reflective of students learning and engagement with the new online teaching methods largely supported online teaching. Students stated that online lectures and practicals were “more efficient”, “easier to learn”, "valuable" and “effective” than the face-to-face sessions". However, ~ 9% (14/150) students suggested that the learning experience can not be replicated online and that face-to-face teaching should continue after COVID-19 pandemic (Table 2).

### Student evaluation: ease of change and future application

Although students mostly agreed that online histopathology teaching was better at integrating the subject into other disciplines of medicine as compared to face-to-face teaching, this received the lowest mean score rating of 4.03/5 (70.6%, 106/150). Most students (≥ 80%) found the switch to online learning for pathology relatively easy compared to other subjects of their medical education (88%, 132/150). Also, students have agreed that online pathology teaching should continue in the future (81%, 122/150) which supports the view for continuing some aspects (especially pre-clinical) of online virtual learning in medical education. Qualitative comments suggested that the online transformation would be “highly beneficial” to future cohorts compared to other aspects of the medical curriculum.

### Impact on student learning outcomes

**Fig. 3:**
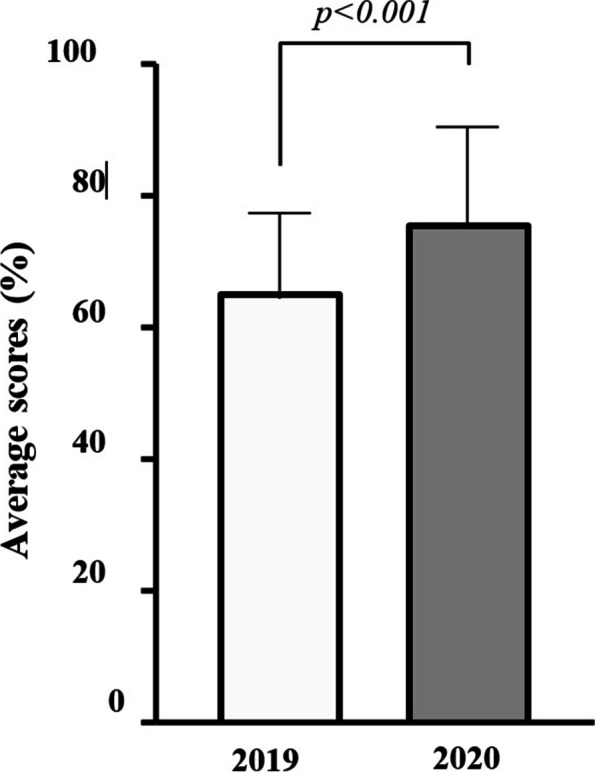
Average marks (%) at the end of year exam within the pathology discipline between 2019 and 2020. Analysis was completed using the Mann–Whitney test, an unpaired non-parametric analysis based on the non-normal distribution of the data according to the Shapiro–Wilk test

Compared to Year-2 medical students who sat their pathology examination in 2019, there was a significant improvement in marks in the 2020 cohort following the switch to online learning and teaching (Fig. 3). The mean ± SD overall marks were increased from 65.36% ± 13.12% in 2019 to 75.83% ± 14.84% in 2020 (*p* < 0.05). The difficulty of each exam between years, as determined by a pre-set minimum standard to pass (sum of minima), was not significantly different (*p* > 0.05) and thus did not appear to account for the improvement in results.

Compared to their performance during the year 1 curriculum (2019), there was no significant difference (*p* = 0.30) at an individual level between scores in 2020 following the transition to online learning (*n* = 215; 2019 [mean ± SD] = 75.94% ± 10.52, 2020 [mean ± SD] = 75.52% ± 15.63).

## Discussion

This study evaluated the effectiveness of online histopathology teaching in medical education during the COVID-19 pandemic. Traditional teaching in pathology combines didactic lectures with practical sessions and utilises a combination of face-to-face microscopy and gross tissue specimens as well as digitised images [4]. The main objective of this study was to determine if switching to an entirely online method of teaching histopathology was well-received by students and was capable of providing students with the adequate knowledge in pathology required for their medical education. Students received lectures entirely online using a visual demonstration of microscopic pathology by means of a virtual microscopy system. This was followed by a series of online practical sessions which required student involvement in answering clinical self-assessment questions to integrate the essential aspects of each disease entity into their medical training. Results from student evaluations showed that this novel method of teaching histopathology to medical students was more engaging and beneficial to student learning than traditional methods and in turn, opens a new avenue for the ongoing use of virtual classrooms and practicals in teaching medical pathology.

In the current global crisis, medical education has been forced to undergo significant changes and has adapted to overcome many hurdles. Online teaching and learning have been a complex but necessary change encountered during the pandemic across many educational platforms [8–10]. This change has not been entirely novel as in recent years, advances in the use of multimedia and technology have seen the gradual incorporation of new, interactive, online learning environments [4, 11]. However, these techniques have been integrated for use in conjunction with traditional learning techniques such as face-to-face lectures and practicals, and in-person microscopy exposure. Granting students access to virtual slides and pathology materials to supplement their face-to-face learning has made a significant impact on student performance and engagement in histopathology, with prior studies demonstrating that digital imaging systems have excellent use in providing students with access to study materials both on and off-campus [12–15].

Although an entirely digitised method of teaching limits a students ability to gain experience and become competent in microscopy techniques, it may open a whole new avenue of opportunity to enable better access to resources, to provide further reach to students who are unable to attend in-person sessions, and to facilitate self-directed learning to best prepare students for their future clinical years. Additional advantages of online pathology teaching are its cost-effectiveness and its ability to create unlimited opportunities to connect with long-distance consultants and lecturers in telepathology [15]. This may facilitate further integration of clinical knowledge into the pre-clinical years of medicine, ultimately benefiting student education. In addition, studies have proven that online teaching has a better impact on tracking student participation and creates a safe environment for ongoing professional development and interactive learning [15, 16].

In this study, we have noted a significant positive learning experience in online pathology sessions as compared to traditional face-to-face teaching. Student evaluations were largely in support of online learning as it was engaging, beneficial to learning, easy to access and interact with, and was an overall better experience than face-to-face sessions. Current studies have only examined the impact of virtual pathology teaching in conjunction with traditional face-to-face learning but have also found these virtual methods to be extremely valuable in improving the student learning experience in pathology [4].

In addition to analysis of student experiences, an exploratory analysis was completed to assess for changes in student performance with the shift to an online pathology curriculum. There was no significant difference (*p* = 0.30) in performance within the same cohort between the first- and second-year curriculum and a significant improvement (*p* < 0.05) in student performance during the second-year curriculum when compared to a prior cohort. These exploratory results appear to indicate that the transition to an online-based curriculum was not associated with any detrimental effects on student performance. These results are in keeping with previous studies that have suggested no significant difference (i.e. no detriment to learning) between students' experience in digital pathology and conventional teaching [4, 17].

The analysis of student performance includes several confounding factors that could impact student performance which could not be controlled given the study design. Although there was no significant difference in the difficulty and standard between exams, as determined by the sum of minima comparisons, we cannot neglect the presence of other factors which may have affected exam performance. The exam was delivered in an entirely new online format and although measures were put in place to prevent and detect cheating, the obvious risk of unethical student behaviour remained. As the proctoring service was also via an online program, it was limited in its ability to detect student misconduct as students were not directly visualised taking their exam. Furthermore, given that students experienced more time at home throughout the year due to restrictions on all university activities, it is possible that students utilised this time to study and/or studied more effectively as in-person classes and travel time were eliminated. In addition, traditional histopathology assessments at Griffith University are conducted across 10–20 stations consisting of 3 questions with 30 s allowed per question and no back-tracking. The online assessment allowed time for reflection, back-tracking and gave students the opportunity to modify answers and return to challenging questions which have never been a feature of in-person assessments in the past. Thus, changes in results or lack thereof cannot solely be attributed to online delivery methods of teaching. Further studies with stricter exam regulations or standardisation are needed to assess the full capacity of virtual histopathology teaching in medical education. Importantly the assessment results indicate that the change to an online mode of delivery did not appear to worsen student performance.

## Conclusions

An entirely online teaching mode for pathology in medical education is associated with improved student learning experiences, positive attitudes towards histology and pathology learning, and better engagement by students. Despite the limitations relating to analysis, the shift to online teaching did not appear to be associated with any detriment to learning outcomes compared to pre-COVID. These findings support the integration of online teaching methods in pathology education and suggest that virtual practical sessions be continued in future (post-COVID). Eventually, this may surpass the need for face-to-face practical sessions due to improved student engagement and performance and the equal distribution of clinically relevant knowledge to all medical students.

## Data Availability

The datasets used and/or analysed during the current study are available from the corresponding author on reasonable request.
